# Supplementary material for: Development of chemokine network inhibitors using combinatorial saturation mutagenesis
Source: Commun Biol. 2025 Apr 3;8:549. doi: 10.1038/s42003-025-07778-6 (PMC11969024; doi:10.1038/s42003-025-07778-6)
Supplement: Supplementary file 4 — Supplementary Data 2 [file 42003_2025_7778_MOESM4_ESM.zip › SupplementaryData3/scripts/xgeo.tar!/./script/analysis/JK/GSE198600_homo_atherosclerosis_plaque_boxplot.pdf]

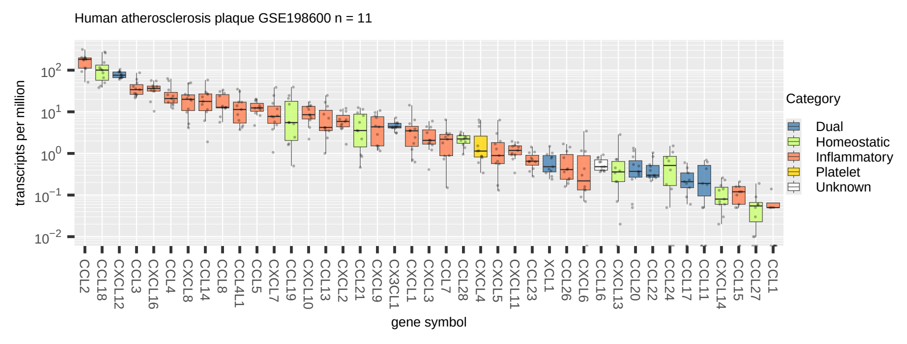

Human atherosclerosis plaque GSE198600 n = 11
transcripts per million
10^2
10^1
10^0
10^-1
10^-2
gene symbol
CCL2
CCL18
CXCL12
CCL3
CXCL16
CCL4
CXCL8
CCL14
CCL8
CCL4L1
CCL5
CXCL7
CCL19
CXCL10
CCL13
CXCL2
CCL21
CXCL9
CX3CL1
CXCL1
CXCL3
CCL7
CCL28
CXCL4
CXCL5
CXCL11
CCL23
XCL1
CCL26
CXCL6
CCL16
CXCL13
CCL20
CCL22
CCL24
CCL17
CCL11
CXCL14
CCL15
CCL27
CCL1
Category
Dual
Homeostatic
Inflammatory
Platelet
Unknown
